# Expanding the scope: multimodal dimensions in aphasia discourse analysis—preliminary findings

**DOI:** 10.3389/fnhum.2024.1419311

**Published:** 2024-09-25

**Authors:** Manaswita Dutta, Bijoyaa Mohapatra

**Affiliations:** ^1^Department of Speech and Hearing Sciences, Portland State University, Portland, OR, United States; ^2^Department of Communication Sciences and Disorders, Louisiana State University, Baton Rouge, LA, United States

**Keywords:** aphasia, discourse, multimodal communication, gestures, connected speech, macrolinguistic quality, meaning-laden gestures, main concept

## Abstract

**Background:**

Aphasia, resulting from acquired brain injury, disrupts language processing and usage, significantly impacting individuals’ social communication and life participation. Given the limitations of traditional assessments in capturing the nuanced challenges faced by individuals with aphasia, this study seeks to explore the potential benefits of integrating multimodal communication elements into discourse analysis to better capture narrative proficiency in this population.

**Objective:**

This study examined how incorporating multimodal communication elements (e.g., physical gestures, writing, drawing) into discourse analysis may affect the narrative outcomes of persons with aphasia compared to those observed using methods that exclude multimodal considerations.

**Methods:**

Participants included individuals with chronic aphasia and age-and education-matched healthy controls who completed a storytelling task—the Bear and the Fly story. Macrolinguistic scores were obtained using verbal-only and multimodal scoring approaches. Additionally, the frequency and type of multimodal communication use during storytelling were examined in relation to aphasia characteristics. Statistical analyses included both within-group and between-group comparisons as well as correlational analyses.

**Results:**

Individuals with aphasia scored significantly higher in terms of their macrolinguistic abilities when multimodal scoring was considered compared to verbal-only scoring. Within the aphasia group, there were prominent differences noted in macrolinguistic scores for both fluent and nonfluent aphasia. Specifically, both groups scored higher on Main Concepts when multimodal scoring was considered, with the nonfluent group demonstrating significantly higher Main Concept and total macrolinguistic rubric scores in multimodal scoring compared to verbal scoring on the storytelling task. Additionally, aphasia severity showed moderate positive correlations with total macrolinguistic scores, indicating that individuals with less severe aphasia tended to produce higher quality narratives. Lastly, although persons with aphasia used different types of nonverbal modalities (i.e., drawing, writing), the use of meaning-laden gestures was most predominant during storytelling, emphasizing the importance of multimodal elements in communication for individuals with aphasia.

**Conclusion:**

Our preliminary study findings underscore the importance of considering multimodal communication in assessing discourse performance among individuals with aphasia. Tailoring assessment approaches based on aphasia subtypes can provide valuable insights into linguistic abilities and inform targeted intervention strategies for improving communication outcomes.

## 1 Introduction

### 1.1 Aphasia and its impact on social communication and life participation

Aphasia, a neurological condition commonly occurring as a result of an acquired brain injury affecting the left cerebral hemisphere, can disrupt the intricate mechanisms of language processing and use (Murray and Clark, 2015). While aphasia primarily impairs linguistic functions, its consequences ripple through various aspects of an individual’s life, most notably in social communication and life participation (Dalemans et al., 2010a,b; Wallace, 2010).

The effects of aphasia on social communication are far-reaching and multifaceted. Language is the cornerstone of human interaction, allowing individuals to express thoughts, feelings, and ideas, as well as to understand and interpret the messages of others. For someone with aphasia, this intricate web of communication becomes disrupted, leading to difficulties in retrieving and forming words, constructing sentences, and comprehending speech. Aphasia can also hinder the formation and maintenance of relationships (Fotiadou et al., 2014). Conversations that were once effortless can become challenging, leading to a sense of isolation, withdrawal, and reduced self-esteem. Friends and family members may struggle to adapt to the communication changes, inadvertently causing further social disconnect (Parr, 2007).

The inability to convey thoughts and emotions effectively may lead to misunderstandings, potentially resulting in feelings of frustration and sadness for both the persons with aphasia and their communication partners (Blom Johansson et al., 2012a,b; Linebaugh et al., 2006). Moreover, aphasia can impact participation in various life activities (Dalemans et al., 2010a,b; Sjöqvist Nätterlund, 2010). Simple tasks like grocery shopping, attending appointments, meeting with friends, or engaging in leisure activities become more daunting. Individuals with aphasia may find it challenging to navigate public spaces, request assistance, or communicate their needs effectively (Howe et al., 2008; Parr, 2007). These barriers can negatively impact their confidence and sense of independence, limiting their involvement in everyday life participation (Hilari, 2011; Michallet et al., 2003). Employment and educational opportunities may also be curtailed due to aphasia’s influence as individuals with aphasia may struggle to perform tasks that involve reading, writing, or verbal communication (O’Halloran et al., 2024; Pike et al., 2017).

### 1.2 Moving beyond discrete impairment-centric assessments: a focus on discourse-level language outcomes

Traditionally, impairment-focused assessments [e.g., Western Aphasia Battery-Revised (Kertesz, 2007)] and discrete tasks such as naming, verbal fluency, auditory comprehension, and sentence repetitions have been most commonly employed to evaluate language impairments in persons with aphasia (Faroqi-Shah and Milman, 2018; Nozari et al., 2010). While these assessments are valuable for pinpointing specific linguistic deficits associated with aphasia, they fall short in providing a comprehensive picture of the multifaceted communication challenges that persons with aphasia face when using language in their everyday communication. Moreover, it has been increasingly recognized that these isolated language tasks lack the sensitivity required to capture the subtle deficits experienced by those with milder forms of aphasia (DeDe and Salis, 2020; Fromm et al., 2017). Relying solely on standardized assessments may fail to identify individuals with these milder profiles of aphasia, resulting in a lack of tailored support and services available to them (Cavanaugh and Haley, 2020).

In line with the International Classification of Functioning, Disability and Health Framework proposed by the World Health Organization (2018), it is crucial that aphasia assessment and rehabilitation approaches extend beyond addressing language deficits at the impairment level (i.e., improvement of spoken and written words and sentences) and take into account the impact of these language impairments on the individual’s daily activities and life participation. It is well agreed upon that the primary objective of aphasia rehabilitation should be to improve everyday communication, thereby supporting persons with aphasia to better function in their day-to-day lives. Consequently, there has been a notable shift within the field towards focusing on connected speech and functional communication outcomes for those with aphasia (e.g., Bryant et al., 2017; Doedens and Meteyard, 2020; Purdy and Wallace, 2016).

In recent years, the analysis of spoken discourse, or language use beyond isolated words and sentences has garnered significant attention in aphasia assessment, treatment, and research (Boyle et al., 2023; Bryant et al., 2017; Casilio et al., 2019; Cruice et al., 2020; Dipper et al., 2021; Dutta et al., 2024b; Kong and Wong, 2018; Mohapatra, 2019; Park et al., 2024; Stark et al., 2021b). Consequently, clinicians and researchers are increasingly integrating spoken discourse analysis into their practice to not only characterize language outcomes but also to target complex language processing and determine the efficacy of language and communication interventions (Kim et al., 2019; Mohapatra and Mohan, 2023; Rider et al., 2008). Evaluating discourse abilities is important as it is more functional compared to decontextualized language tasks (e.g., picture naming, sentence repetition) and captures communication challenges experienced by individuals with aphasia that negatively impact their activity and life participation.

Spoken discourse analysis allows the evaluation of language in terms of microstructural (e.g., grammatical complexity, lexical diversity), macrostructural (i.e., coherence, amount of meaningful content conveyed), and social and pragmatic aspects (Dutta, 2020). A variety of tasks can be used to elicit discourse samples including single picture and picture sequence descriptions, storytelling/retelling, interviews, or structured and unstructured conversations with familiar or unfamiliar communication partners (Leaman and Archer, 2023; Richardson and Dalton, 2016).

Storytelling is a complex task because it requires the integration of multiple extralinguistic cognitive processes including attention, memory, and executive functioning, and linguistic skills (Büttner-Kunert et al., 2022; Cahana-Amitay and Jenkins, 2018; Cannizzaro and Coelho, 2013; Mohapatra, 2020). During storytelling, speakers must extract conceptual information from stories or fables, integrate it with their general knowledge, and sequence this information in a logically and syntactically organized manner to produce meaningful stories (Peach and Wong, 2004). Linguistically, storytelling involves processing at both microlinguistic (within-utterance) and macrolinguistic (across-utterance) levels (Andreetta et al., 2012). This reflects the ability to make appropriate lexical selections, use a rich and diverse vocabulary, construct complex sentences, and employ narrative structures to guide the content and flow of the story. Additionally, it involves the creative use of language to engage the audience (Labov and Waletzky, 1997; Reilly, 2004). Cognitively, speakers must infer the motivation and intention for protagonists’ actions, draw logical relations between events and align these events with the theme of the story and evaluate the meaning or significance of events in the story, making storytelling a sophisticated and multifaceted task (Sherratt, 2007; Ska et al., 2009).

Although monologic storytelling tasks are not fully representative of functional communication and lack the interactivity of real-world conversations (e.g., Armstrong et al., 2011; Beeke et al., 2003; Dipper et al., 2018; Mayer and Murray, 2003; Leaman and Edmonds, 2021, 2023; Leaman and Archer, 2023), they provide a clinically feasible means for clinicians to evaluate an individual’s spontaneous language use across multiple linguistic levels (e.g., phonological, lexical, morphological, syntactic, semantic) (Bryant et al., 2017; Marini et al., 2011). Furthermore, these tasks provide insights into the complex interactions between extralinguistic cognition and language skills, serving as a valuable complement to formalized aphasia assessment tools [Dipper et al., 2021; see Dutta et al. (2024a) and Hill et al. (2018) for discussions on the associations between cognitive deficits and discourse impairment in brain injury]. Performance on storytelling tasks offers guidance regarding areas that should be prioritized in both assessment and treatment. For instance, narratives produced by individuals with aphasia may be analyzed quantitively for impairments in structural processes (phonological processing, lexical selection, grammatical construction), informativeness, and semantic connections between the utterances (coherence, both local and global) (Linnik et al., 2022; Marini et al., 2011; Richardson and Dalton, 2016). Further, individuals who experience difficulties in planning and narrating stories in a meaningful and coherent manner may be suspected of having an executive planning impairment (Cannizzaro and Coelho, 2013; Greenslade et al., 2020). Similarly, difficulties in recalling key information and characters from a story may indicate working memory challenges, impacting their story informativeness and efficiency (Cahana-Amitay and Jenkins, 2018). In terms of treatment planning, if individuals with aphasia demonstrate impaired performance on storytelling tasks, therapy approaches such as script training may be considered to train them in narrating personally relevant stories (e.g., stroke story, recent vacation). Additionally, contemporary discourse treatments such as the Narrative and Discourse Intervention in Aphasia (NADIIA; Whitworth et al., 2015; Whitworth, 2024) can be implemented to specifically target executive functioning skills including initiation, planning, organization, working memory, cognitive flexibility, and self-monitoring in the context of discourse (Dipper et al., 2024).

### 1.3 Multimodal communication in aphasia

In the quest for effective communication strategies for individuals with aphasia, researchers and clinicians have turned their attention to a dynamic approach known as multimodal communication which recognizes the importance of integrating alternative modalities such as writing, drawing, gestures, and communication boards alongside speech to facilitate meaningful interactions (Holland, 2021, 1982; Purdy and Wallace, 2016; van der Meulen et al., 2010). Aphasia literature underscores the value of multimodal communication as a versatile and empowering tool in managing the impact of this disorder.

In the realm of aphasia, where traditional verbal communication may be compromised, the integration of multiple communication modalities offers a diverse toolkit that complements and augments speech. Supplementary modalities such as gesturing, writing, music/melody/non-speech sounds, or drawing provide visual and physical avenues for expression (Doedens and Meteyard, 2020; Pierce et al., 2019). For instance, when individuals with aphasia experience significant challenges with verbal production, writing can be a strength and can be leveraged as an alternative modality for everyday communication (Clausen and Besson, 2003). This can include using a sophisticated alternative and augmentative communication (AAC) device or a basic low-tech LCD writing and drawing tablet. AAC systems are physical or digital tools that incorporate images, symbols, or words to support language expression (e.g., Jacobs et al., 2004; Nicholas et al., 2011; Purdy and Dietz, 2010). These tools serve as a bridge between a person’s internal thoughts and their external communication and offer a structured and organized means of communication, allowing those with aphasia to point to or select relevant symbols to convey their intended message, even when speech is compromised (Lasker and Garrett, 2006; Nicholas et al., 2011). Similarly, gesture use is frequently reported in aphasia (Purdy et al., 1994; Sekine et al., 2013). These individuals can use hand movements to meaningfully convey concepts, including specific objects and actions to either augment the conceptual content already expressed in speech or add new conceptual content that is not in speech, enabling clearer communication with others (Glosser et al., 1986; Pritchard et al., 2015; Sekine et al., 2013).

One of the strengths of multimodal communication lies in its adaptability to the diverse profiles of individuals with aphasia. Language deficits vary widely, and what works effectively for one person might differ for another. Multimodal approaches can be tailored to suit an individual’s specific strengths, preferences, and communication goals (Lasker et al., 2005; Pierce et al., 2019). This customization enhances the likelihood of successful communication outcomes and empowers individuals to participate more actively in social interactions and daily activities. The benefits of multimodal communication extend beyond the individual with aphasia to their communication partners and broader social circles. Caregivers, family members, friends, and healthcare professionals can learn to interpret and respond to the alternative modalities being used, promoting more effective and empathetic interactions (Pierce et al., 2019). Therefore, multimodal communication facilitates shared communication that goes beyond traditional speech, bridging communication gaps and potentially fostering deeper connections while reducing feelings of isolation and frustration among individuals with aphasia.

In the exploration of macrolinguistic qualities within storytelling, it becomes evident that the richness and depth of narratives extend far beyond verbal expression alone (Pritchard et al., 2015; Sekine et al., 2013). Discourse analysis in aphasia has traditionally focused on verbal output, neglecting the myriad of other communication modalities that individuals with aphasia often utilize. This oversight dismisses the significance of gestures, drawings, non-speech sounds, and other nonverbal forms of expression that play a crucial role in aiding their verbal productions. These supplementary modes of communication, although vital in conveying narrative details, have been excluded from conventional scoring systems, potentially skewing the evaluation of individuals’ storytelling abilities. The failure to account for these diverse forms of expression may have resulted in undervaluing the storytelling capacities of individuals with aphasia on specific variables.

Most aphasia research has focused on multimodal communication in therapy contexts, with limited empirical investigations in language assessment (Cunningham and Ward, 2003; Purdy et al., 1994; Purdy and Wallace, 2016). In aphasia assessment, some measures of communicative success are available (Azios et al., 2022; Garrett and Huth, 2002). For example, Leaman and Edmonds (2019) have developed a macrolinguistic multimodal measure called Communicative Success which measures how successfully an individual communicates in context on a 4-point rating scale. On this scale, a rating of 4 (“entirely successful”) indicates that the message was clearly communicated, while a rating of 1 (“not successful”) indicates that the message was unclear. This measure takes into account both verbal and nonverbal behaviors, as well as the communication partner’s response to the person with aphasia’s turn. The Communicative Success measure has demonstrated adequate test–retest stability, inter-rater reliability, and sensitivity in structured, elicited monologues and unstructured conversation conditions (Leaman and Edmonds, 2019, 2021, 2023). Similarly, Kurland et al. (2023, 2024) introduced the Brief Assessment of Transactional Success (BATS) wherein people with aphasia retold stories after watching or listening to short video/audio stimuli and engaged in conversations to co-construct the stories with their communication partners. Following the conversational exchange about the retold stories, the measure was applied to evaluate the conversation partner’s monologic story retelling based on what they learned from the person with aphasia. Participant responses are coded for macrolinguistic quality such as Main Concepts and topic similarity. In terms of psychometrics, the BATS demonstrated acceptable test–retest stability and inter-rater reliability (Kurland et al., 2024). Prutting and Kittchner (1987) developed the Pragmatic Protocol that provided an overall communicative index for school-age children, adolescents, and adults across 30 pragmatic aspects of language including verbal aspects (e.g., turn taking, topic introduction and maintenance, lexical selection, cohesion), and paralinguistic elements (e.g., vocal quality, prosody) in addition to nonverbal aspects (e.g., body posture, gesture use, facial expression, eye gaze). The protocol can be completed after observing individuals engage in spontaneous interactions with a communicative partner and each pragmatic aspect of the protocol can be assessed as appropriate, inappropriate, or not observed. Collectively, the abovementioned measures that incorporate both verbal and nonverbal elements have been studied in monologic discourse and conversational contexts. However, no studies have directly compared discourse scoring procedures with and without the inclusion of multimodal criteria, nor examined how this could potentially influence the quality of discourse output among those with aphasia. As such, the current study explores the scope to include alternative communication forms into existing monologic discourse scoring systems, thus enabling a more comprehensive and accurate assessment of the strengths and weaknesses within aphasic discourse.

### 1.4 Aim

The study aims to investigate how incorporating multimodal communication elements (e.g., physical gestures, writing, drawing) into discourse analysis may change the narrative outcomes captured from persons with aphasia during a structured storytelling task, when compared to methods that do not include multimodal considerations. Specifically, we compared narrative outcomes of persons with aphasia with healthy controls and analyzed differences in macrolinguistic quality between verbal-only and multimodal scoring, both across and within groups as well as based on aphasia characteristics. Additionally, we examined the distribution of multimodal communication subtypes among speakers with aphasia. Our hypothesis posited that by incorporating alternative modalities, individuals with aphasia would demonstrate improved macrolinguistic scores in their storytelling performances compared to when scoring is based solely on verbal communication. Conversely, we anticipated these scoring differences to be less pronounced among healthy controls.

## 2 Materials and method

### 2.1 Participants

We conducted a comparative analysis of the discourse performances of persons with aphasia (PWA) and age-and education-matched healthy controls (HC) using the Bear and the Fly storytelling task (PWA *n* = 22; HC *n* = 24 from Dutta, 2020; Dutta et al., 2023, 2024a). All participants were native English speakers and had normal or corrected-to-normal vision and hearing. See Table 1 for participant demographics. To note, this work was part of a larger research project investigating the relationship between cognitive skills (specifically working memory and executive functioning) and narrative discourse abilities in aphasia (Dutta et al., 2024a). All participants completed the storytelling task along with a battery of executive functioning tasks. The study was approved by the Indiana University Institutional Review Board and all participants provided written informed consent before data collection.

**Table 1:** Demographic characteristics of study participants.

Variable	Persons with aphasia (*n* = 22) Mean ± SD	Healthy controls (*n* = 24) Mean ± SD	Group comparison statistic
No. of Participants	24	22	
Age	63.68 ± 9.3	61.16 ± 9.40	U = 85, *p* = 0.586
Education	16.63 ± 2.90	17 ± 2.8	U = 93.5, *p* = 0.865
Sex	14/8	6/18	
Times post brain injury (months)	81.52 ± 85.26	–	
WAB-AQ	74.40 ± 17	–	
MoCA	2013	27 ± 1	

Group differences in age and education were assessed using Mann–Whitney U tests.

SD, Standard Deviation; WAB-R AQ, Western Aphasia Battery-Revised Aphasia Quotient (Kertesz, 2007); MoCA, Montreal Cognitive Assessment (Nasreddine et al., 2005).

The inclusion criteria for the aphasia group were (a) people who developed chronic aphasia resulting from an acquired brain injury (i.e., stroke, traumatic brain injury, encephalitis), and (b) a diagnosis of aphasia on the Western Aphasia Battery–Revised (WAB-R; an aphasia quotient [AQ] score < 93.8) or as per clinician’s judgment of language abilities. The exclusion criteria were (a) a history of dementia or any progressive neurological conditions (e.g., primary progressive aphasia), and (b) any history of drug abuse or alcoholism. The inclusion criteria for the healthy control group were (a) no neurological condition (e.g., stroke, head injury), and (b) normal cognitive functioning on the Montreal Cognitive Assessment (MoCA; MoCA score ≥ 26 out of 30; Nasreddine et al., 2005). The exclusion criteria were (a) developmental language disorders (e.g., dyslexia), and (b) any history of drug abuse or alcoholism.

The aphasia group included individuals with a range of aphasia types and severities with WAB-AQ scores ranging between 37.8 and 100. In terms of aphasia types, participants exhibited anomic aphasia (*n* = 7), Broca’s aphasia (*n* = 8), conduction aphasia (*n* = 3), and transcortical motor aphasia (*n* = 1), with severity spanning from very mild to severe. Three individuals with scores above the WAB-AQ cut-off (i.e., scores higher than 93.8; classified as not aphasic by the WAB) were included in the study based on the ongoing language challenges these individuals experienced in their daily communication, notably impacting their discourse-level output (Fromm et al., 2017; Richardson et al., 2021). Additionally, ten participants in the aphasia group demonstrated mild to severe apraxia of speech on the Apraxia Battery for Adults (ABA-2; Dabul, 2000); most persons with aphasia had no limb apraxia with two individuals demonstrating mild impairment. The cognition assessment revealed that individuals with aphasia performed significantly worse than the healthy control group on various executive functioning tasks, including those assessing auditory-verbal and visuospatial working memory, inhibition, cognitive flexibility, verbal and figural fluency, initiation, planning, reasoning, problem-solving, and self-monitoring. Both verbal and nonverbal tasks posed challenges for the aphasia group, with verbal executive functioning tasks being particularly difficult. Additionally, the aphasia group had slower reaction times on timed tasks compared to controls. There was considerable variability in executive functioning impairments within the aphasia group, with not all participants showing deficits. For detailed assessment methods and results, see Dutta et al. (2023). See Supplementary Table S1 for demographic information and neuropsychological assessment scores from the battery of cognition and language tests administered for individual participants.

### 2.2 Procedures

#### 2.2.1 Discourse sample elicitation

Participants told the Bear and the Fly story from a wordless picture book (Winter, 1976). The story narratives were elicited by initially providing color drawings in a wordless picture book to the participants, who were allowed to view these sequential pictures for as long as they wished. After the book was taken away, participants were asked to tell the story in their own words. No prior narration was provided by the examiner. Since the main focus of the larger research project (Dutta et al., 2024a) was to investigate the role of executive functioning in discourse, we instructed participants to tell the story without the aid of the book. No time restrictions were set, and participants were prompted to use complete sentences and include as much detail as possible in their story. Prior to beginning the task, all participants were offered a blank sheet of paper and a writing utensil. They were asked to verbally narrate their story but were encouraged to use any communication modality (i.e., AAC, writing, drawing) to supplement their storytelling. All participants were evaluated individually in a quiet laboratory setting, and the language samples were audio and video recorded for further analysis.

#### 2.2.2 Discourse transcription, scoring, and analysis

The narrative samples were transcribed orthographically by the first author and trained graduate research assistants (RAs). Following transcription, each narrative sample was segmented into individual utterances, which were defined as “a complete thought, usually expressed by connecting groups of words, which is separated from other utterances on the basis of content, intonation, contour, and/or pausing” (Shewan and Henderson, 1988, p. 124). Any disagreements in scoring or transcription were resolved through discussion. These language samples were then scored using a macrolinguistic scoring rubric developed by Loughnane et al. (2016) and Loughnane and Murray (2018), as referenced by Dutta (2020) and Dutta et al. (2024a). The rubric evaluates the following components (see Supplementary material S2 for the rubric):

- Main Concepts: The total number of main ideas produced related to the story (total possible score = 15).
- Organization: Providing relevant information, being topic-centered, using appropriate cohesion and length (total possible score = 5).
- Language use: The use of mental state and describing words (e.g., whacked, frustrated) to add information to the story (total possible score = 10).

Two scoring systems were compared: (1) traditional verbal scoring focusing exclusively on verbal output and (2) a modified system that took into account nonverbal modalities like gestures, drawing, and writing. Author MD completed the verbal-only scoring for all samples; Trained graduate and undergraduate research assistants scored the Bear and Fly samples using the modified multimodal scoring approach. Video recordings of each participant’s language sample were reviewed by the coder. Coders were instructed to watch each video at least once before beginning to code. Following this initial viewing, coders watched the recordings again to identify and mark all main story concepts produced by the participant. They also had access to the transcribed and segmented samples to verify these main concepts. Each main concept was assigned a score of 1 if present or 0 if absent. Coders were encouraged to rewatch the video to assign scores for organization and to list any mental state or descriptive words used under the language use section. Author MD conducted training sessions for the assigned RAs on the updated scoring methodology. Following an explanation of the procedures, author MD and the RAs collaboratively coded two sample cases. Any queries regarding the scoring protocols were addressed, and demonstrations were provided on how to document instances of verbal communication breakdowns and the use of multiple communication modes. Subsequently, the trained RAs were tasked with scoring the discourse samples using the macrolinguistic rubric, keeping in mind the multimodal approach. Specifically, they were instructed to award points if the participant was able to successfully communicate the story elements through speech or any other communication modality utilized.

For the modified scoring system, similar coding procedures as described for the traditional macrolinguistic rubric scoring were followed. However, in this approach, coders reviewed the video recordings of the discourse samples and were instructed to assign a point for each relevant main concept or descriptive word produced with and without the use of supplementary communication modalities. During the discourse task, participants were encouraged to use any communication modality available to them (e.g., gestures, drawing), in addition to the pen and paper provided by the examiner. Some individuals with aphasia had personal AAC devices that they used in aphasia therapy and other communication settings, and they were permitted to use their devices during the storytelling task. Participants with and without aphasia frequently used multimodal communication, primarily co-speech gestures, to supplement verbal communication, even when no breakdown occurred. However, we only counted instances of alternative modality use during verbal communication breakdowns. Anecdotally, the use of co-speech gestures did not significantly impact macrolinguistic scores. For instance, if a participant verbally conveyed a story concept, it would receive a score of 1 regardless of the presence of supplementary co-speech gestures.

Verbal communication breakdowns referred to difficulties or failures experienced by participants in effectively verbalizing the story. These breakdowns were objectively identified by coders when there were clear indications of them during the storytelling process. Among persons with aphasia, these included instances when they experienced word-finding difficulties (e.g., difficulty finding the words ‘fly swatter’) and/or where the participant was unable to convey key main concepts such as the bear hitting the mom on the head, or the mother bear being injured. These were noted if the speaker was unable to retrieve a word within 5 seconds, visibly showed difficulties in finding a word, substituted an incorrect word for the target (e.g., ‘switch’ for ‘swatter’), or made remarks like ‘I cannot say the word.’ Additionally, some individuals also used gestures to depict actions, such as swatting, alongside phrases like ‘what do you call it.’ These were all considered signs of communication breakdowns. In some cases of word retrieval failures, participants switched to a different modality (e.g., gestures, writing), which subsequently facilitated access and enabled verbal production of the target word. These instances were still coded for communication breakdowns and alternative modality use. Further, even when an individual quickly and effectively used a gesture in place of a word—whether to prevent a breakdown or simply out of preference (e.g., to convey the story elements of father bear swatting the mother bear and she was hit on the head, one participant produced “he hit the mother and she [gestures mother bear was knocked off])—these were counted due to the absence of speech, especially since verbal language was primarily used to convey the rest of the story. Instances of verbal communication breakdowns and subsequent alternative modality use were marked in the ELAN software (EUDICO Linguistic Annotator; Max Planck Institute for Psycholinguistics, 2002; www.mpi.nl/tools/). Coders had access to written transcriptions of the discourse samples while coding in ELAN, which provided a textual reference to ensure all story content was accurately identified and marked. Following the coding of nonverbal modalities, we tallied the types and frequencies of nonverbal modalities used.

The gesture coding initially involved categorizing hand movements into gestures and non-gesture movements. Non-communication gestures (e.g., touching one’s own face or hair, changing posture or hand position) were excluded due to their lack of meaningful relevance to the content of the storytelling task. Each detected gesture produced was coded using the guidelines outlined by Rose et al. (2013) and Preisig et al. (2018). See Table 2 for descriptions and examples of each gesture type. We specifically coded for instances when participants used alternative modalities to communicate story concepts when they experienced language breakdowns (e.g., inability to produce story ideas or word finding difficulties). For example, persons with aphasia wrote down nouns (e.g., ‘bear,’ ‘chair’) or used gestures to indicate actions such as ‘swatting,’ or ‘knocked off,’ when they could not verbally produce these words when telling the story. The identified gestures were further categorized as: (1) meaning-laden gestures; (2) abstract gestures; and (3) unclassifiable gestures. Meaning-laden gestures included gestures that conveyed meanings related to the semantic content of the narrated story. These included deictic gestures, emblems, letter gestures, number gestures, pointing to self, and iconic gestures which represent concrete actions, events, or objects as though the speaker is observing it from afar (observer viewpoint) or as though he is the character/object itself (character viewpoint; McNeill, 1992). Abstract gestures conveyed abstract meaning (e.g., gesture to depict the concept of time) or did not convey any specific meaning. Types of abstract gestures included beat, metaphoric, referential, and time gestures. Some produced gestures that did not fit within the classification of meaning-laden or abstract gestures were classified as unclassifiable gestures.

**Table 2:** Gesture categories and examples adapted from Rose et al. (2013), Preisig et al. (2018), and van Nispen et al. (2017).

Type	Description	Examples
*1) Meaning-laden gestures*		
Iconic character viewpoint (CVPT)	The speaker employs their own body to portray a specific action, event, or object, embodying the character or object itself	To illustrate someone running, they might swing their arms back and forth, mimicking the act of running
Iconic observer viewpoint (OVPT)	The speaker portrays a tangible action, event, or object as if they are viewing it from a distance. For instance, to illustrate someone running, the speaker might trace their index finger through the air from left to right, as if observing the scene as a distant onlooker	To depict a bird flying in the sky, the speaker might raise their hand high and slowly move it across
Deictic	The speaker signals a tangible reference within the physical surroundings	Refers to a specific object such as a picture book, a piece of real clothing, or a real furniture
Emblem	Form and significance are determined by the customs of particular groups and are often comprehensible without verbal communication	Using a gesture of forming a circle with the thumb and pointer finger to signal “OK” or shaking the head from side to side indicating disagreement or negation
Pantomime	Comprises of two or more CVPT gestures that occur consecutively within a single gesture unit. Regardless of the number of CVPT gestures performed in sequence, they are considered as one pantomime	In a pantomime depicting rowing a boat, the speaker might combine the actions of gripping an oar, pulling it through the water, and leaning back with each stroke
Letter	Actions linked to tracing letters in the air, on a desk, or on one’s thigh using either an empty hand or fingers	The speaker spelling words while narrating a story by tracing the letters on their desk with their index finger
Number	Utilizes the speaker’s fingers to represent numerical values	The speaker depicting the number of main characters in the story using fingers
Pointing to self	The speaker gestures towards their own body, typically their chest, to indicate themselves	When narrating an important life event, the speaker points to their chest when referring to their personal experiences
*2) Abstract gestures*		
Referential	Assigns referents, such as objects, places, or characters in a story, into the space in front of the speaker when no physical object is present. The hand gesture typically involves pointing or mimicking holding an object	While telling a story about a distant castle, the speaker gestures as if holding a majestic structure in the air before them, visually placing the castle within the narrative space
Beat	Gestures characterized by repetitive movements synchronized with speech production, devoid of explicit meaning	While recounting a story, the speaker unconsciously gestures with their hands in a repetitive manner, accompanying the flow of their narrative without conveying specific information through the gestures themselves
Metaphoric	Depicts an abstract concept, such as knowledge, justice, language, or the genre of the narrative, typically represented with a cup-shaped hand gesture	In a discussion about equality, the speaker employs a cupped hand gesture to symbolize the abstract notion of justice, visually conveying the idea of fairness and impartiality
Time	Designates a specific area to represent a point in time, such as the past (behind the body) or the future (in front of the body)	During storytelling, the speaker may gesture towards the area behind them to evoke events from the past, and then gesture towards the space in front of them to suggest future outcomes in the story
*3) Unclassifiable gestures*	Movements or actions that defy categorization within the abovementioned established frameworks of gesture analysis. They may lack clear meaning, context, or recognizable patterns that would allow them to be easily classified alongside other gestures	

#### 2.2.3 Inter-rater and intra-rater reliability

Inter-and intra-rater reliability for transcription, segmentation, and macrolinguistic rubric scoring were determined by randomly selecting 20% of the samples (i.e., five samples from the aphasia and control groups respectively). The selected samples were re-transcribed and re-scored by two additional trained undergraduate and graduate RAs who were not involved in the original scoring as well as authors MD and BM. Inter-rater reliability for segmentation, identification of multimodal communication use, and macrolinguistic rubric scoring were assessed with a two-way random average absolute agreement intraclass correlation coefficient (ICC) model. Intra-rater reliability was evaluated using a two-way mixed average absolute agreement ICC model. Interpretations were made and reported in accordance with the descriptive definitions of coefficient strength provided by Koo and Li (2016) based on the 95% confident interval of the ICC estimates. Reliability on transcriptions was evaluated using point-to-point agreement.

Inter-rater transcription agreements measured by percent agreement between raters was 91%. For utterance segmentation, good reliability was noted (ICC = 0.759; 95% CI range = 0.703; 966). Inter-rater reliability for the transcription of words (ICC = 0.992; 95% CI range = 0.990, 0.999) was determined to be excellent.

Two assigned raters reviewed all selected narrative samples and identified instances of nonverbal communication use. Only the instances of alternative modality use related to verbal communication breakdowns were identified and tallied. The inter-rater reliability coefficient was determined to be 0.925 (95% confidence interval [CI] = 0.864, 0.978) indicating excellent reliability.

Inter-rater and intra-rater reliability ICC values for the verbal-only scoring variables ranged between 0.781–0.947 (95% CI range = 0.581, 0.986) and 0.644–0.976 (95% CI range = 0.533, 0.997) showing moderate to excellent reliability, respectively. In the multimodal scoring, inter-and intra-rater reliability ICC values for the macrolinguistic rubric discourse variables ranged between 0.756–0.996 (95% CI range = 0.689, 0.999) and 0.778–0.951 (95% CI = 0.629, 0.999), demonstrating good to excellent reliability. The inter-and intra-rater reliability ICC values for the macrolinguistic rubric discourse measures are displayed in Table 3.

**Table 3:** Inter-rater and intra-rater reliability intraclass coefficient (ICC) values with confidence interval ranges for the macrolinguistic rubric measures across both verbal-only and multimodal scoring.

Macrolinguistic variable	Inter-rater ICC value	Intra-rater ICC value
Verbal-only scoring		
Main concepts	0.947 [0.781, 0.944]	0.975 [0.848, 0.996]
Organization	0.781 [0.681, 0.962]	0.991 [0.991, 0.944]
Language use	0.798 [0.581, 0.843]	0.644 [0.533, 0.884]
Total macrolinguistic rubric score	0.864 [0.864, 0.986]	0.976 [0.857, 0.997]
Multimodal scoring		
Main concepts	0.996 [0.929, 0.999]	0.996 [0.968, 0.999]
Organization	0.984 [0.843, 0.998]	0.991 [0.944, 0.999]
Language use	0.756 [0.689, 0.900]	0.778 [0.629, 0.899]
Total macrolinguistic rubric score	0.988 [0.898, 0.999]	0.992 [0.941, 0.999]

### 2.3 Statistical analysis

All statistical procedures were conducted using the Statistical Package for the Social Sciences Version 27.0 for Windows. A post-hoc power analysis was conducted using G*Power (Faul et al., 2009), employing a medium effect size (*d* = 0.50) and an alpha level of 0.05. The analysis revealed a power of 0.82, indicating satisfactory statistical power. The Shapiro–Wilk normality test was used to determine if a variable was normally distributed. Differences in discourse performance between groups were assessed with one-way analysis of variance (ANOVA). Within each group, differences in the two scoring systems were examined using paired samples *t*-tests.

To evaluate how the macrolinguistic scores varied with aphasia severity, correlation analysis was used. Participant scores obtained through verbal-only and multimodal scoring were correlated with WAB-AQ scores. Separate paired samples t-tests were employed for fluent and nonfluent aphasia subgroups to compare verbal-only and multimodal scores within each subgroup. Lastly, the types of alternative communication modalities employed by persons with aphasia during storytelling were tallied. Further, associations between gesture use and aphasia severity (i.e., WAB-AQ scores) were explored using correlation analyses. Cohen’s *d* effect sizes were calculated for all inferential statistical analyses with thresholds of small (0.2), medium (0.5), and large (0.8) used to interpret effect sizes (Cohen, 1988). Multiple comparison corrections using the False Discovery Rate (FDR) method (Benjamini and Hochberg, 1995) were applied to group differences and correlational analyses. The *p*-values reported in the results section and tables reflect these adjusted significance levels.

## 3 Results

### 3.1 Between-group comparisons of verbal-only and multimodal scoring

Individuals with aphasia scored lower than healthy controls on all macrolinguistic variables using both verbal-only and multimodal scoring indicating compromised macrolinguistic quality of their narrative output. Specifically, persons in the aphasia group demonstrated significantly fewer main concepts, poorer organization, and less efficient use of mental state and describing words compared to the healthy control group in the verbal-only scoring as well as the multimodal scoring (see Table 4).

**Table 4:** Results from the between-group comparisons of macrolinguistic scores for persons with aphasia and healthy controls when considering verbal-only vs. multimodal scoring of Bear and the Fly story.

Macrolinguistic variable	Aphasia (*n* = 22) Mean ± SD	Healthy controls (*n* = 24) Mean ± SD	Between-group comparison statistic	Cohen’s *d*
Verbal-only scoring				
Main Concepts*	6.50 ± 4.81	12.04 ± 3.30	*F* (1,44) = 21.003, *p* < 0.001	4.09
Organization*	2.86 ± 1.90	4.75 ± 1.03	*F* (1,44) = 17.777, *p* < 0.001	1.51
Language use*	2.68 ± 2.16	5.04 ± 2.69	*F* (1,44) = 10.584, *p* = 0.002	0.194
Total macrolinguistic rubric score*	12.04 ± 7.37	21.25 ± 5.51	*F* (1,44) = 23.237, *p* < 0.001	3.46
Multimodal scoring				
Main concepts*	8.04 ± 4.11	11.79 ± 3.20	*F* (1,44) = 11.995, *p* = 0.001	3.66
Organization*	2.72 ± 1.69	4.45 ± 0.93	*F* (1,44) = 18.842, *p* < 0.001	1.35
Language use*	0.95 ± 1.25	4.45 ± 3.03	*F* (1,44) = 25.316, *p* < 0.001	0.365
Total macrolinguistic rubric score*	11.72 ± 6.12	20.70 ± 4.75	*F* (1,44) = 31.113, *p* < 0.001	4.14

Group differences were assessed using a one-way ANOVA.

* Statistically significant difference at *p* < 0.05 (corrected for multiple comparisons using False Discovery Rate); SD, Standard Deviation.

### 3.2 Within-group comparisons of verbal-only and multimodal scoring

Within the aphasia group (Table 5), individuals scored significantly higher when multimodality was considered: Persons with aphasia exhibited higher scores for main concepts and overall macrolinguistic quality compared to verbal-only scoring on the Bear and the Fly story. For the healthy control group, significant within-group differences were observed in total macrolinguistic rubric scores, while other narrative variables did not show significant differences.

**Table 5:** Results from the within-group comparisons of macrolinguistic scores for persons with aphasia when considering verbal-only vs. multimodal scoring of Bear and the Fly story.

Macrolinguistic variable	Verbal-only scoring Mean ± SD	Multimodal scoring Mean ± SD	Within-group comparison statistic	Cohen’s *d*
Aphasia (*n* = 22)				
Main Concepts*	6.50 ± 4.81	8.04 ± 4.11	*t* (21) = −4.543, *p* < 0.001	1.59
Organization	2.86 ± 1.90	2.72 ± 1.69	*t* (21) = 0.767, *p* = 0.601	0.83
Language use	2.68 ± 2.16	2.94 ± 1.25	*t* (21) = 4.490, *p* = 0.750	1.80
Total macrolinguistic rubric score*	11.72 ± 6.12	12.04 ± 7.37	*t* (21) = 0.572, *p* < 0.001	2.60
Healthy controls (*n* = 24)				
Main concepts	12.04 ± 3.30	11.79 ± 3.20	*t* (23) = 1.466, *p* = 0.121	0.65
Organization	4.75 ± 1.03	4.45 ± 0.93	*t* (23) = 2.290, *p* = 0.064	0.62
Language use	4.45 ± 3.03	5.04 ± 0.54	*t* (21) = 2.509, *p* = 0.064	1.13
Total macrolinguistic rubric score*	20.70 ± 0.97	21.25 ± 5.51	*t* (23) = 1.026, *p* < 0.001	2.58

Within-group differences evaluated using paired samples *t*-tests.

* Statistically significant difference at *p* < 0.05 (corrected for multiple comparisons using False Discovery Rate); SD, Standard Deviation.

### 3.3 Differences in macrolinguistic scores based on aphasia characteristics

#### 3.3.1 Based on aphasia severity

Aphasia severity, measured by WAB-AQ scores was positively correlated with all macrolinguistic rubric variables; these correlations were observed for both verbal-only and multimodal scoring. WAB-AQ scores were moderately correlated with the number of main concepts produced (r_verbal_ = 0.617; r_multimodal_ = 0.545), organization (r_verbal_ = 0.607; r_multimodal_ = 0.650), and total macrolinguistic scores (r_verbal_ = 0.627; r_multimodal_ = 0.588) of those with aphasia during their storytelling of the Bear and the Fly story (all *p* < 0.05; corrected for multiple comparisons using FDR). No significant correlations were noted between WAB-AQ and the language use variable in either modalities. Regardless of the scoring approach, individuals with less severe aphasia produced higher macrolinguistic scores when they told the story.

#### 3.3.2 Based on aphasia subtypes

For the verbal scoring, individuals with fluent aphasia scored higher on main concepts (*F* (1, 20) = 13.750, *p* = 0.001), organization (*F* (1, 20) = 13.360, *p* = 0.002), and total macrolinguistic rubric scores (*F* (1, 20) = 15.487, *p* < 0.001) compared to those with nonfluent aphasia. No difference was noted for language use (*F* (1, 20) = 0.049, *p* = 0.827). Similar results were obtained for the multimodal scoring: Individuals in the fluent group demonstrated higher scores for main concepts (*F* (1, 20) = 81.181, *p* = 0.010), organization (*F* (1, 20) = 14.201, *p* = 0.003), and total macrolinguistic rubric scores (*F* (1, 20) = 10.733, *p* = 0.006) compared to the nonfluent group whereas no group difference was found for language use (*F* (1, 20) = 0.797, *p* = 0.383) (all significance levels corrected for multiple comparisons using FDR).

Both fluent and nonfluent groups scored significantly higher on macrolinguistic variables when multimodal scoring was considered: Within the fluent group (*n* = 13), the number of main concepts were significantly higher when multimodal scoring was considered compared to verbal-only scoring. The nonfluent group (*n* = 9) demonstrated significantly higher scores for main concepts, language use, and total macrolinguistic quality in the multimodality scoring versus verbal-only scoring showing large effect sizes. The differences in scores across modalities was evidently larger for the nonfluent compared to the fluent group. No significant group differences were noted for organization (see Table 6).

**Table 6:** Macrolinguistic scores (verbal-only and multimodal) based on aphasia subtypes for the Bear and the Fly story.

Macrolinguistic variable	Verbal-only scoring Mean ± SD	Multimodal scoring Mean ± SD	Within-group comparison statistic	Cohen’s *d*
Fluent aphasia (*n* = 13)				
Main concepts*	9.00 ± 3.51	9.84 ± 3.76	0.030*	1.14
Organization	3.84 ± 1.57	3.61 ± 1.19	0.169	0.83
Language use	2.76 ± 2.31	1.15 ± 0.42	0.278	0.54
Total macrolinguistic rubric score	14.61 ± 5.09	15.61 ± 5.47	0.102	1.68
Nonfluent aphasia (*n* = 9)				
Main concepts*	2.88 ± 4.19	5.44 ± 3.20	<0.001*	1.66
Organization	1.44 ± 1.42	1.44 ± 1.50	0.500	0.86
Language use*	0.67 ± 0.70	2.55 ± 2.06	0.034	1.70
Total macrolinguistic rubric score*	6.88 ± 6.88	7.55 ± 5.15	<0.001*	3.42

Within-group differences evaluated using paired samples *t*-tests.

* Statistically significant difference at *p* < 0.05 (corrected for multiple comparisons using False Discovery Rate); SD, Standard Deviation.

### 3.4 Types of multimodal communication use during storytelling among speakers with aphasia

Regarding the types of alternative communication modalities used by participants during verbal communication breakdowns in storytelling, we found that individuals with aphasia most commonly used gestures (95.5%), followed by writing (2%), drawing (1.5%), and AAC (0.8%).

For gesture use, as illustrated in Table 7, those within the aphasia group produced a greater proportion of meaning-laden gestures while telling the Bear and the Fly story (49% of total gestures produced). These were largely characterized by the use of iconic-character and iconic-observer viewpoint gestures. In contrast, they produced a fewer proportion of abstract gestures (30% of total gestures produced), most of which were classified as referential gestures. Approximately 20% of the gestures for each storytelling were unclassifiable. We further explored the correlations between aphasia severity (i.e., WAB-AQ scores) and the frequency of meaning-laden (*r* = 0.128, *p* = 0.285) and abstract gestures (*r* = −0.093, *p* = 0.341) produced, and the results were not remarkable.

**Table 7:** Incidence of verbal communication breakdowns as well as the types and frequency of gestures utilized during storytelling among persons with aphasia and healthy controls.

	Aphasia (*n* = 22) Mean ± SD	Healthy controls (*n* = 24) Mean ± SD
Verbal communication breakdowns	9.14 ± 6.01	0.40 ± 0.51
Iconic character viewpoint (CVPT)	2.29 ± 3.01	0.04 ± 0.20
Iconic observer viewpoint (OVPT)	5.05 ± 4.12	0.08 ± 0.28
Deictic	0.17 ± 0.38	0
Emblem	0	0
Pantomime	0	0
Letter	0.31 ± 0.63	0
Number	0	0
*Total meaning-laden gestures*	*7.09 ± 6.05*	*0.12* ± 0.33
Referential	2.89 ± 2.82	0.08 ± 0.28
Beat	0.62 ± 0.96	0
Metaphoric	0	0
Time	0	0
*Total abstract gestures*	*2.73 ± 2.97*	*0.08 ± 0.28*
*Unclassifiable*	*1.29 ± 1.61*	*0*

The gesture categories outlined by Rose et al. (2013) and Sekine et al. (2013) were followed for coding in the current study; SD, Standard Deviation.

## 4 Discussion

The current study examined narrative discourse abilities of persons with aphasia and age-and education-matched HC, focusing on their performance on a structured storytelling task. Building upon the understanding of aphasia’s significant impact on individuals’ engagement in daily activities (Boyle, 2011; Davidson et al., 2008), this study critically examines the outcomes to determine the effects of multimodal communication components, including physical gestures, drawing, and writing, on the overall macrolinguistic proficiency of persons with aphasia. Specifically, the study employed both verbal-only and multimodal scoring systems to assess macrolinguistic abilities in individuals with and without aphasia, providing a comprehensive understanding of language production beyond traditional verbal output. This preliminary exploration extends beyond traditional impairment-and speech production-centric assessment approaches, providing insights into the use of multimodal communication use during structured storytelling and its contribution to macrolinguistic discourse processes, as well as the implications for clinical practice and intervention strategies in aphasia rehabilitation.

### 4.1 What are the macrolinguistic quality differences observed in storytelling between persons with aphasia and healthy controls?

The findings reveal significant differences between individuals with aphasia and healthy controls across different macrolinguistic variables. Regardless of the scoring method, those with aphasia consistently scored lower than controls, indicating compromised macrolinguistic quality in their narrative output. Notably, speakers with aphasia demonstrated fewer main concepts, poorer organization, and less efficient language use compared to HC; the group comparisons revealed relatively larger effect sizes for the main concepts and medium effect sizes for organization in both modalities. These findings underscore the profound impact of aphasia on the ability to construct semantically rich and organized narratives, reflecting challenges in conveying complex story structures and details accurately (Linnik et al., 2016; Richardson et al., 2021) (Mohan and Mohapatra, In review)^1^. Consistent with Cahana-Amitay and Jenkins (2018) and Kuvač Kraljević et al. (2023), individuals in the aphasia group faced challenges in producing key story details and presenting them coherently. Besides impairments in linguistic abilities, these difficulties can be linked to poor cognitive performance. Specifically, as observed in our previous study (Dutta et al., 2024a), challenges in tasks assessing verbal fluency, working memory, planning, and slower processing speed were strongly associated with poor macrolinguistic narrative quality. Although the use of alternative modalities was beneficial, their overall macrolinguistic quality remained significantly lower compared to age-and education-matched healthy adults.

### 4.2 Do macrolinguistic scores differ between verbal-only and multimodal scoring approaches in storytelling within each group?

Within the aphasia group, the comparison between verbal-only and multimodal scoring revealed significant differences in narrative performance, with multimodal scoring yielding higher scores for these individuals. These preliminary findings suggest that incorporating nonverbal modalities enhances overall macrolinguistic quality and provides alternative means of expression for persons with aphasia during structured discourse tasks (Richardson and Dalton, 2016). The statistically significant improvement in scores with multimodal assessment underscores the importance of considering multiple communication channels in assessing discourse abilities and tailoring interventions to address the unique communication needs of those with aphasia.

Our findings demonstrate that multimodal scoring can effectively highlight competence in individuals with aphasia, even when verbal performance is compromised. During the storytelling task, participants demonstrated the ability to utilize nonverbal modalities to convey main characters and story ideas when faced with difficulties in verbal expression. Our multimodal scoring approach revealed communication strengths that might not be fully captured through verbal assessments alone. Traditional discourse scoring approaches that predominantly rely on verbal output tend to overlook the narrative abilities of individuals with aphasia, despite their ability to successfully communicate ideas using modalities other than speech.

In contrast, within the healthy control group, significant differences were found between verbal-only and multimodal scoring on total macrolinguistic scores with no significant differences on other variables. The healthy adults primarily relied on verbal expression and experienced fewer communication breakdowns in their narrative construction compared to individuals in the aphasia group. Therefore, they did not need to use alternative modalities during their storytelling as much as persons with aphasia. This finding suggests that while nonverbal modalities may enhance narrative performance in individuals with aphasia, they may not offer the same benefits for healthy control participants considering the strengths in their verbal communication.

### 4.3 Is there variation between multimodal and verbal scoring based on aphasia characteristics?

The analysis revealed significant moderate correlations between aphasia severity and macrolinguistic scores obtained through both verbal-only and multimodal assessments on the Bear and Fly story. Specifically, in line with Richardson et al. (2021) and Ulatowska et al. (1983), individuals with greater aphasia severity tended to exhibit lower macrolinguistic scores in both assessment approaches. This finding underscores the impact of aphasia on overall discourse proficiency, irrespective of the discourse assessment modality used. This is contrary to prior research by Hogrefe et al. (2012) who have documented that individuals with more severe aphasia can in fact convey more information using alternative modalities such as gestures during narrative story retelling. Consistent with Hogrefe and colleagues’ findings, however, we found no correlation between performance on standardized aphasia testing (i.e., the WAB-R) and any gestural indices specifically. The standardized format and specific instructions of the WAB tasks might constrain individuals with aphasia from spontaneously incorporating gestures into their verbal responses.

Our analysis revealed that aphasia severity was significantly correlated with storytelling macrostructural scores, irrespective of the scoring approach employed. However, WAB-AQ showed only moderate correlations with main concepts, organization, and total macrolinguistic scores. Similar to Lasker and Garrett (2006), who found performance on the WAB to elucidate patterns of communication strategy use among people with aphasia, our preliminary study findings showed moderate correlations between Aphasia Quotient and macrolinguistic performance, however aphasia severity was not associated with the extent of communication strategy use across different modalities in this population. Therefore, it is crucial to recognize that evaluation of communication should not be limited to formal aphasia assessments, as these tasks may underestimate the frequency and variety of alternative modalities used by individuals with aphasia, failing to capture their full communicative repertoire (Dutta et al., 2024a). This highlights the need for discourse-level assessments to capture the nuances of gesture use in connected speech (de Beer et al., 2017). Unlike verbal-only discourse scoring assessments, which primarily capture linguistic deficits, multimodal scoring approaches and assessments offer a more comprehensive understanding of individuals’ communication abilities by considering a broader range of additional expressive modalities that can enhance communicative functions among those with aphasia (Pierce et al., 2019; van der Meulen et al., 2010). Therefore, multimodal communication modalities should be considered more routinely in assessing storytelling proficiency among individuals with aphasia.

In terms of aphasia subtypes, persons with fluent aphasia exhibited significantly higher overall macrolinguistic quality in their storytelling compared to those with nonfluent aphasia. Specifically, they achieved higher scores in total number of main concepts produced and showed better discourse organization in both verbal-only and multimodal scoring assessments. When the scoring types were compared, individuals with both fluent and nonfluent aphasia were shown to produce significantly more main concepts related to the stories when multimodal scoring was incorporated versus verbal-scoring alone. This demonstrates a clear advantage for individuals with different types of aphasia when modalities other than speech are considered in the scoring systems. Although individuals with fluent aphasia demonstrated better organization compared to those with nonfluent aphasia, incorporation of nonverbal modalities in the scoring did not change the scores in this domain. Maintaining organization in narratives may likely depend more on the integrity of verbal productions, with gestures or other modalities playing a minor role in enhancing story coherence. Alternatively, the frequency and types of nonverbal modalities utilized during the storytelling task did not significantly affect organization scores, likely due to the limited number of story episodes in the Bear and Fly task. With a longer, more detailed story, the potential benefits of incorporating multiple modalities alongside speech could become more pronounced.

Interestingly, when performances were examined within each aphasia subtype, as seen in previous studies (Herrmann et al., 1988; Nicholas et al., 2011; Ozturk and Özçalışkan, 2024), speakers with nonfluent aphasia utilized multiple modalities more effectively and showed greater benefits during storytelling compared to those with fluent aphasia. Individual-level analysis revealed that during storytelling, individuals in the nonfluent aphasia subgroup frequently used specific language (i.e., content words) in response to the discourse stimuli, albeit with limited linguistic complexity. This was also reflected in their nonverbal communication modality use. To illustrate, the differences between the average main concept and total macrolinguistic rubric scores of the nonfluent group across modalities were more pronounced compared to the fluent group (Table 6). This is further evidenced by the larger effect sizes observed: The Cohen’s *d* values for total macrolinguistic rubric scores across multimodal and verbal-only scoring in the nonfluent group was 3.42, as opposed to 1.68 in the fluent group. Anecdotally, these participants more frequently gestured, wrote, and drew to indicate key characters and actions to convey the main elements in their stories (e.g., used specific nouns like ‘bear,’ ‘chair’). As an example, some individuals used swatting gesture to supplement their speech and convey the central idea of ‘the father bear attempts to swat the fly’ when experiencing difficulties with word retrieval. In the multimodal scoring process, the scorer carefully considered the conveyed concept and intended meaning by the person with aphasia, acknowledging this benefit of using nonverbal modalities in augmenting their communication abilities. Consequently, their expressions were counted as main concepts rather than absent ones. Collectively, our findings add to a limited pool of existing evaluation tools that encompass multimodality for people with aphasia and evaluate aspects of multimodal behavior (eye gaze, gestures, facial expressions) in spontaneous speech including the Pragmatic Protocol (Prutting and Kittchner, 1987), the Communicative Success measure (Leaman and Edmonds, 2019, 2023 2024), and Brief Assessment of Transactional Success (Kurland et al., 2023, 2024). The incorporation of such standalone tools alongside modifications to existing discourse scoring protocols in aphasia assessment enhances understanding of communication abilities beyond traditional linguistic measures alone.

### 4.4 How can the use of multimodal communication be characterized in persons with aphasia during structured storytelling?

When evaluating the types of nonverbal modalities used by persons with aphasia during storytelling, our results, consistent with Rose et al. (2013), indicated that gestures were most commonly used. Among gestures, individuals within the aphasia group predominantly utilized meaning-laden gestures, such as iconic character and iconic-observer viewpoint gestures when telling the story. In line with Stark and Cofoid (2022), this finding suggests that people with aphasia frequently use gestural cues to convey narrative content, potentially compensating for verbal language deficits. The consistent use of these gestures underscores their importance as one of the primary modes of communication for persons with aphasia when language is impaired (Akhavan et al., 2018). It is important to highlight that although in this study we only counted instances of alternative modality use during verbal communication breakdowns for discourse analysis coding, we anecdotally observed that the gestures produced by persons with aphasia were used during and outside of communication breakdowns. That is, gestures were used either to clarify information in their verbal output (i.e., co-speech gestures; Kistner et al., 2019) or to convey information about the stories in the absence of speech (most commonly in case of word finding difficulties and difficulties producing the main concept verbally). Specifically, iconic gestures, which bear meaning closely tied to the semantic content of their speech, emerged as a prevalent tool employed by individuals with aphasia, particularly evident during storytelling. Our preliminary findings suggest that gestures improve communication success in aphasia and provide support for theoretical models such as the Sketch Model (De Ruiter, 2000) and the Interface model (De Kita and Özyürek, 2003), that argue that gesture and speech originate from a shared communicative intention but proceed to production via separate channels (De Ruiter, 2000; Hogrefe et al., 2012). Compatible with these accounts, our study results revealed that gestures served as a communicative tool for persons with aphasia compared to the control group during storytelling; gestures played a more flexible and compensatory role when speech was restricted during storytelling (De Ruiter, 2000; De Ruiter and de Beer, 2013). Further, producing gestures also facilitated word production in some participants with aphasia (Rose and Douglas, 2001).

Moreover, the occurrence of deictic, emblematic, pantomime, and letter gestures was minimal among the aphasia group. This is in contrast to previous studies by Sekine et al. (2013) and Rose et al. (2013) who observed that individuals used emblems and pantomime gestures frequently, albeit this was noted when they engaged in conversational discourse. The infrequent use of these gesture types by the aphasia group in the current study suggest limited opportunities to employ these specific communication modalities within the structured story telling task and controlled testing environment which may have constrained their use compared to a natural setting. Participants in the current study also exhibited a lower frequency of abstract gestures, with referential gestures being the most prevalent within this category. Although these gesture types were observed less, they still contributed significantly to the overall multimodal communication during storytelling. Collectively, our findings demonstrate that people with aphasia employ both concrete and abstract gestures to enhance the richness and complexity of their narratives.

Interestingly, in contrast to Mol et al. (2018), our results did not indicate an association between types of gestures used and aphasia severity, as measured by WAB-AQ scores. This suggests that gesture use may not be directly influenced by the severity of aphasia but rather reflects individual differences in communication preferences and abilities among participants including factors like limb apraxia, semantic processing, and extralinguistic cognitive skills such as potentially attention, working memory, and executive functioning (Cocks et al., 2018; Hogrefe et al., 2012; Mohapatra and Laures-Gore, 2021; Purdy and Koch, 2006; Stark et al., 2021a). These factors are important to consider because they commonly co-occur with aphasia (Dutta et al., 2023; Mohapatra and Marshall, 2019). Nonetheless, the lack of available data precluded a definitive confirmation of this hypothesis in the current study, emphasizing the need for further investigation.

While persons with aphasia also utilized other modalities such as writing, drawing, and AAC, these were relatively limited in frequency. Despite reminders to use other modalities when needed, we observed that some individuals in the aphasia group did not use these tools consistently. They experienced difficulties to independently switch to a different communication modality (e.g., writing, using gestures) when verbal communication breakdowns occurred, possibly indicating impairments in cognitive flexibility and self-monitoring. While some participants attempted to use pen and paper or AAC systems, their ability to successfully communicate story elements through nonverbal modalities was often limited. For example, one participant relied on abstract or vague gestures during word-finding difficulties, resulting in reduced communication quality. Considering that nonverbal communication may vary with different discourse tasks (Ozturk and Özçalışkan, 2024; Stark and Cofoid, 2022), it is plausible that the storytelling task used in the current study did not provide sufficient opportunities to elicit diverse nonverbal communication modalities. Recognizing the advantages of alternative communication modes for people with aphasia, it is crucial to explore their patterns of use in various discourse contexts beyond traditional and structured assessments.

### 4.5 What are the clinical implications of the study findings for assessment and intervention strategies in aphasia therapy?

The findings of this study have implications for clinical assessment and intervention in aphasia. Integrating multimodal assessment approaches can provide a more comprehensive evaluation of language abilities and inform individualized treatment plans tailored to the unique communication needs of persons with aphasia. Traditionally, discourse assessment has predominantly focused on verbal expression, neglecting information conveyed through nonverbal communication modalities like gestures, writing, and drawing. Individuals living with aphasia frequently utilize these alternative modes to supplement their verbal communication. The initial results of our current investigation highlight the efficacy of integrating a modified scoring system that recognizes the significance of such alternative modalities such as physical gestures, drawing, and writing in conveying essential concepts. This inclusive approach significantly improved the macrolinguistic scores of persons with aphasia, offering a more holistic evaluation of their narrative proficiency. Relying solely on verbal scoring would disregard these valuable dimensions of discourse, potentially underestimating narrative abilities of those with aphasia. To characterize gesture and multimodal use during discourse productions, clinicians are encouraged to use ready-to-use tools such as the City Gesture Checklist (Caute et al., 2021) or incorporate measures such as the Communicative Success measure to evaluate how gesture use may facilitate speech during structured discourse production and conversations. Additionally, discourse assessment tasks provide an accurate context to examine the use of gestures. Overall, clinicians are encouraged to expand beyond conventional discourse scoring methods and adopt the modified approach delineated in the present study to thoroughly assess communication outcomes among persons with aphasia.

Moreover, interventions targeting the integration of nonverbal modalities into narrative production may enhance communication effectiveness among individuals with aphasia. Specifically, those with aphasia can be trained to use multimodal communication strategies (e.g., writing, AAC, or using gesture types that are more effective in conveying meaning) through treatments such as Promoting Aphasics’ Communication Effectiveness (Carlomagno et al., 1991), or more contemporary approaches such as Multimodal Aphasia Therapy (M-MAT; Pierce et al., 2024), or the Multimodal Communication Treatment (Purdy and Wallace, 2016) to improve communicative success.

Overall, this study contributes to our understanding of the complex interplay between verbal and nonverbal communication in aphasia and underscores the importance of adopting holistic assessment approaches to capture the richness and complexity of language production in this population. Further research is warranted to elucidate the mechanisms underlying the relationship between language impairment and multimodal communication and to develop effective interventions that leverage these modalities to optimize communication outcomes for individuals with aphasia.

### 4.6 Limitations and future directions

The current study has several limitations that warrant consideration. Firstly, due to its preliminary nature, our investigation was constrained by a small sample size, which may have limited the generalizability of our findings. However, expanding the participant pool with more diverse aphasia profiles would allow comparisons of different aphasia types and severities and yield more robust estimates of multimodal utilization during storytelling tasks.

Moreover, our examination of multimodal communication abilities was confined to a single discourse task. Although our study presents new findings showing that macrolinguistic scores of those with aphasia improve when alternative modalities are considered, results from these monologue tasks cannot be used to inform individuals’ performances in everyday communication or conversation. Thus, future research should explore the frequency, variety, and purpose of such alternative modalities in this population, particularly within more everyday interactive social contexts such as conversations. Another limitation was that the samples evaluated in the current study did not elicit a wide range of multimodal strategy use apart from gestures (e.g., drawings, AAC devices). This could potentially be a resultant of the nature of the task used and instructions provided or limited access to story-specific vocabulary. In the current study, we employed a structured elicited storytelling task. Although participants were encouraged to use alternative modalities when narrating the story, the length of the story number of episodes were limited which could have potentially restricted the demonstration of multiple modality use. Future research can explore multimodality use in other discourse contexts (e.g., procedural discourse, conversations) and with more explicit instructions. In the current study, we used a macrolinguistic rubric that evaluated main concepts and organization. Although this measure has been employed in previously published research and has been found to be an accurate metric in evaluating macrolinguistic quality in a range of acquired brain injury etiologies (e.g., Loughnane and Murray, 2018; Dutta, 2020; Dutta et al., 2024a), it is critical to note that there is no normative data available for this measure and it has not yet been evaluated for test–retest stability, which should be acknowledged as important limitations of the study.

Main Concept has serious limitations as a measure for understanding discourse production and more importantly successful communication. For instance, one speaker might accurately express the concepts but also include a high amount of irrelevant information or jargon, resulting in a lower overall communication success. Conversely, another speaker might produce the same number of Main Concepts without using unnecessary, non-meaningful language, leading to a much higher success rate. This discrepancy could further impact the sequencing of the main information, causing breakdowns in the speaker’s expressive language and consequently hindering the listener’s ability to follow the story (Hameister and Nickels, 2018). Therefore, to evaluate the macrolinguistic quality of discourse more comprehensively, future studies should incorporate additional measures, such as Global Coherence (Wright et al., 2010; Wright et al., 2013) or Main Concept, Sequencing, and Story Grammar (MSSG; Richardson et al., 2021), to capture macrolinguistic functioning more accurately. This approach would offer a better understanding of discourse production and successful communication.

## 5 Conclusion

Consistent with previous research, our preliminary findings from this study show that people with aphasia exhibit lower macrolinguistic quality in their discourse productions compared to healthy adults. Notably, our results reveal that persons with aphasia score higher in terms of their narrative abilities when their multimodal communication use is considered in the discourse scoring. Participants in the current study employed a variety of modalities, with gestures combined with speech being the most prevalent, to convey crucial details about the stories. When considering these alternative nonverbal elements in the scoring process, there was a notable increase in the overall information content and macrolinguistic quality. This emphasizes the significance of expanding assessment approaches beyond the verbal modality alone and recognizing and integrating multimodal aspects when evaluating discourse abilities in individuals with aphasia.

## Data Availability

Raw data will be made available on request from the corresponding author.

## Footnotes

^1^Mohan, R., and Mohapatra, B. (In review). Conversational discourse in individuals with aphasia during a group collaborative storytelling program.
