# Supplementary material for: Expanding the scope: multimodal dimensions in aphasia discourse analysis—preliminary findings
Source: Front Hum Neurosci. 2024 Sep 25;18:1419311. doi: 10.3389/fnhum.2024.1419311 (PMC11461255; doi:10.3389/fnhum.2024.1419311)
Supplement: Supplementary file 2 [file Table_2.docx]

**Supplementary Material 2.** Macrolinguistic Narrative Analysis Rubric for the Bear and the Fly story (Loughnane et al., 2016; Loughnane & Murray, 2018).

| ***Participant______________ Scored by________________ Date______________*** | | |
| --- | --- | --- |
| ***Narrative Scoring Rubric- Bear and Fly*** | | |
| **Main Concept** | **Organization** | **Language** |
| \| Family sitting to eat \|  \| \| --- \| --- \| \| Fly appears \|  \| \| FB attempts to swat fly \|  \| \| Food spills \|  \| \| Fly keeps moving \|  \| \| MB hit on head \|  \| \| MB injured \|  \| \| BB smacked on head \|  \| \| BB injured \|  \| \| Fly lands on dog \|  \| \| FB swats dog \|  \| \| Dog injured \|  \| \| Puts chair on table \|  \| \| Falls off \|  \| \| Fly leaves \|  \| | - Begins with a concise introduction of the experience *(examples include: The bear family…, “characters” were sitting around the table eating…)* - Ends with a concise conclusion of the experience *(examples include: The end, and the fly flew away leaving everyone behind…)* - Describes interactions in a topic centered manner (relative to the topic). *If indicates extraneous or inaccurate details more than twice, do not check box.* - Details/Elaboration appropriate – measured by number of utterances between 40-60. - Appropriate referencing – none or only one general use of “he,” “she,” and “they” – must reference the character in the previous sentence in order to receive a point *(if adult makes more than two referencing errors they do not receive this point).* | - Mental state* *(for example, "He's looking' like he's teasing him." or "He is furious" or direct quotes, she was like, "you are crazy!")* - Mental state - Mental state - Mental state - Mental state - Detail/describing word* *(describe or add information to the story; e.g., whammied, flatten, buzzing, soggy, smack!, feast, irritating, unconscious)* - Detail/describing word - Detail/describing word - Detail/describing word - Detail/describing word   *don’t count repeats |
| ______/15 = _______ % | ______/5 = _______ % _________/10 = ___________% | |
|  | **TOTAL ______/30= ________%** | |
